# Probabilistic projections of the stability of small tidal inlets at century time scale using a reduced complexity approach

**DOI:** 10.1038/s41598-021-01945-5

**Published:** 2021-11-25

**Authors:** Trang Minh Duong, Roshanka Ranasinghe, David P. Callaghan

**Affiliations:** 1 Department of Coastal & Urban Risk & Resilience, IHE Delft Institute for Water Education, P.O. Box 3015, 2601 DA Delft, The Netherlands; 2 Department of Water Engineering & Management, University of Twente, P.O. Box 217, 7500 AE Enschede, The Netherlands; 3 Harbour, Coastal and Offshore Engineering, Deltares, P.O. Box 177, 2600 MH Delft, The Netherlands; 4 School of Civil Engineering, The University of Queensland, Brisbane, QLD 4072 Australia

**Keywords:** Climate change, Natural hazards, Ocean sciences

## Abstract

Climate change is widely expected to affect the thousands of small tidal inlets (STIs) dotting the global coastline. To properly inform effective adaptation strategies for the coastal areas in the vicinity of these inlets, it is necessary to know the temporal evolution of inlet stability over climate change time scales (50–100 years). As available numerical models are unable to perform continuous morphodynamic simulations at such time scales, here we develop and pilot a fast, probabilistic, reduced complexity model (RAPSTA – RAPid assessment tool of inlet STAbility) that can also quantify forcing uncertainties. RAPSTA accounts for the key physical processes governing STI stability and for climate change driven variations in system forcing. The model is very fast, providing a 100 year projection in less than 3 seconds. RAPSTA is demonstrated here at 3 STIs, representing the 3 main Types of STIs; Permanently open, locationally stable inlet (Type 1); Permanently open, alongshore migrating inlet (Type 2); Seasonally/Intermittently open, locationally stable inlet (Type 3). Model applications under a high greenhouse gas emissions scenario (RCP 8.5), accounting for forcing uncertainties, show that while the Type 1 STI will not change type over the twenty-first century, the Type 2 inlet may change into a more unstable Type 3 system around mid-century, and the Type 3 STI may change into a less unstable Type 2 system in about 20 years from now, further changing into a stable Type 1 STI around mid-century. These projections underscore the need for future adaptation strategies to remain flexible.

## Introduction

Tidal inlets are a common feature all over the world’s coastline^1–9^. Coastlines adjacent to these inlets, known as inlet-interrupted coasts^10,11^, despite being generally very dynamic, have for centuries attracted humans and supported many human activities, including waterfront developments, recreation, fishing, navigation and sand mining^2,8,10,12^. Changes to the inlet or the adjacent coast could therefore impact some or all of these human activities, leading to substantial socio-economic losses, and in some situations, threaten the safety of coastal communities^8–10^. Several recent studies have shown that, due to climate change, such system changes are all but inevitable over the twenty-first century^13,14^, and effective adaptation strategies therefore need to be developed and implemented. To properly inform such adaptation strategies, long term (~ 50–100 years) projections of how inlets may change in future, including quantification of the uncertainty associated in these projections, are required. This study aims to take one small step forward in this regard.

Unlike uninterrupted coasts that are governed only by oceanic processes such as tides, waves and mean sea level (MSL), inlet-interrupted coasts are also governed by terrestrial processes such as riverflow and fluvial sediment^6,8–10,14–18^. Therefore, projected climate change driven variations in any of these drivers can lead to many negative physical impacts on inlet-interrupted coasts, such as shoreline retreat adjacent to the inlet, erosion of estuary margin shorelines, inundation of low lying areas along estuary margins, estuarine eutrophication, and toxic algal blooms etc^17^. At a more fundamental level, climate change driven changes in system forcing may affect the stability of the inlet itself, potentially even leading to a change in “inlet Type” (e.g. permanently open, intermittently open, alongshore migrating).

There are three main classes of tidal inlets: geological origin (also referred to as drowned river valleys), littoral origin (e.g. barrier islands), and hydrological origin (e.g. estuary or river connected to the sea)^19^. Although several previous studies have investigated climate change impacts on very large tidal inlet/basin systems falling into any of these categories (e.g. Dissanayake et al.^20^; van der Wegen^21^; van der Wegen et al.^22^; Lodder et al.^23^), the nature and magnitude of climate change impacts on the more commonly found small tidal inlet/estuary systems, falling within the hydrological origin inlet class, remain less well understood, with only a handful of studies focussing on this aspect (e.g. Ranasinghe et al.^10^; Duong et al.^13,24^; Bamunawala et al.^8,9^). These relatively small estuaries/lagoons, which are also known as “bar-built” or “barrier” estuaries (hereafter referred to as Small Tidal Inlets or STIs for convenience) are commonly found along microtidal, wave dominated mainland coasts, comprising about 50% of the world’s coastline^10^. Following Duong et al.’s^6^ definition, here, STIs are taken to be systems that generally have little or no intertidal flats or ebb tidal deltas. These systems are usually separated from the ocean by a sand spit that is connected to the mainland at one end, in contrast to barrier islands where the sand barrier is completely separated from the mainland by a lagoon. STI systems are further defined^6^ as systems that contain inlet channels that are less than 500 m in width, which are connected to estuaries/lagoons with surface areas less than 50 km^2^ and average depths less than 10 m.

Duong et al.^6^ identifies 3 main STI Types:

- Permanently open, locationally stable inlets (Type 1).
- Permanently open, alongshore migrating inlets (Type 2).
- Seasonally/Intermittently open, locationally stable inlets (Type 3).

While the exact number of STIs present around the world is unknown, this number is likely to run into thousands with predominant occurrence in tropical, sub-tropical and temperate regions (e.g. West and South Africa, and SW/SE Australia, India, Sri Lanka, Vietnam, South America (Brazil), Florida (USA)). Only the number of Type 3 STIs around the world is reported to be close 1500^7^. STIs are particularly common in developing countries where data availability is generally poor (i.e. data poor environments) and community resilience to coastal change and the capacity to adapt is rather low compared to that in developed countries.

### Governing processes

Under contemporary conditions, inlet stability is governed primarily by two processes: (1) nearshore sediment (littoral) transport in the vicinity of the inlet, and (2) flow through the inlet during the ebb phase of the tide^25^. The latter will not only include the ebb tidal prism (volume of water flowing out of the estuary/lagoon due to tidal forcing alone) but also riverflow effects. For convenience, hereon, the ebb flow due to the combined effect of both of these processes will be referred to as the tidal prism.

Inlet stability has traditionally been defined using the Bruun criterion *r* = *P/M*, where *P* = tidal prism (m^3^) and *M* = annual littoral transport (m^3^/year)^25^. Linkages between the Bruun criterion and Inlet Type were quantified by Duong et al.^13^ as shown in Table 1.

**Table 1:** Classification scheme for inlet Type and stability conditions (Reproduced from Duong et al.^13^, all rights reserved).

Inlet Type	*r* = *P*/*M*	Bruun classification
Type 1	> 150	Good
	100–150	Fair
	50–100	Fair to Poor
	20–50	Poor
Type 2	10–20	Unstable (open and migrating)
Type 2/3	5–10	Unstable (migrating or intermittently closing)
Type 3	0–5	Unstable (intermittently closing)

The most severe climate change impact on a given STI would be a change in STI Type. This could potentially affect all or most economic and social activities centred on the STI that had developed over time based on the expectation that the general morphological behaviour of the STI will remain unchanged. For example, if a Type 1 system (permanently open, locationally stable), of which the lagoon is used as an anchorage for sea going vessels, changes into a Type 3 system (intermittently open, locationally stable), it may no longer be possible to operate the anchorage. A less severe, but still potentially very socio-economically damaging climate change impact would be a significant change in the level of stability of an STI (as per the Bruun inlet stability criterion *r*), while its Type remains unchanged. For example, if the level of stability of the same example Type 1 STI changes from ‘good’ (*r* > 150) to ‘poor’ (20 < *r* < 50), although it will still remain a Type 1 inlet, navigation through the inlet might become difficult and perilous, thus seriously compromising its continued functionality as an efficient anchorage.

### Modelling climate change impacts on STIs

The most attractive way to assess climate change driven morphodynamic impacts on STIs would be to use a state-of-the-art process based model such as *Delft3D* or *Mike 21* to simulate their morphodynamics over the entirety of the twenty-first century under a range of climate scenarios, while accounting for the uncertainties in projected sea level rise (SLR), wave conditions and riverflows. However, at present, both model limitations and computational demand do not allow such long term simulations of process based coastal area morphodynamic models with concurrent tide, wave and riverflow forcing that also capture seasonal signatures in wave and riverflow forcing^17,26^. The only attempt with process based models to gain insights into climate change impacts on STIs themselves is reported by Duong et al.^13,24^, who used a strategic snap-shot modelling approach employing *Delft3D*. In this approach, first *Delft3D* is validated at a given study site (1 year simulation), and then the validated model is applied with projected future forcing (SLR, waves, riverflow) at the target future time period (1 year simulation at for e.g. mid-century, end-century), while aggregated physical formulations are used to update the morphology in between the two time periods in an offline fashion. While this snap-shot approach can provide useful insights into the end-state of a STI after a certain period of time, it does not provide a complete picture of how the STI will evolve over the full period of interest. However, following the adaptation pathways philosophy^27,28^, knowledge of how systems evolve through time is necessary to be able to develop and implement the right adaptation strategies at the right times as we move along the twenty-first century. This would typically require continuous century scale model morphodynamic simulations of inlet-interrupted coasts, which have to date only been undertaken with physics based, reduced complexity models such as SMIC^10^ or G-SMIC^8,9,14^. However, while these models are able to provide projections of shoreline change near inlets, they do not provide detailed information of the time evolution of inlet Type and stability, knowledge of which is essential for developing effective adaptation strategies for fragile STI systems. To address this need, here we present a probabilistic, reduced complexity model that can provide continuous 100 year-long simulations of the inlet stability condition of STIs (as defined above) with concurrent tide, wave and riverflow forcing (including seasonal variations in wave and riverflow forcing), while taking into account future forcing uncertainty through the model’s probabilistic framing. The model is demonstrated here at 3 STIs, each representing one of the 3 main Types of STIs found around the world, for a high-end greenhouse gas emissions scenario following RCP 8.5.

## The model

**Figure 1:**
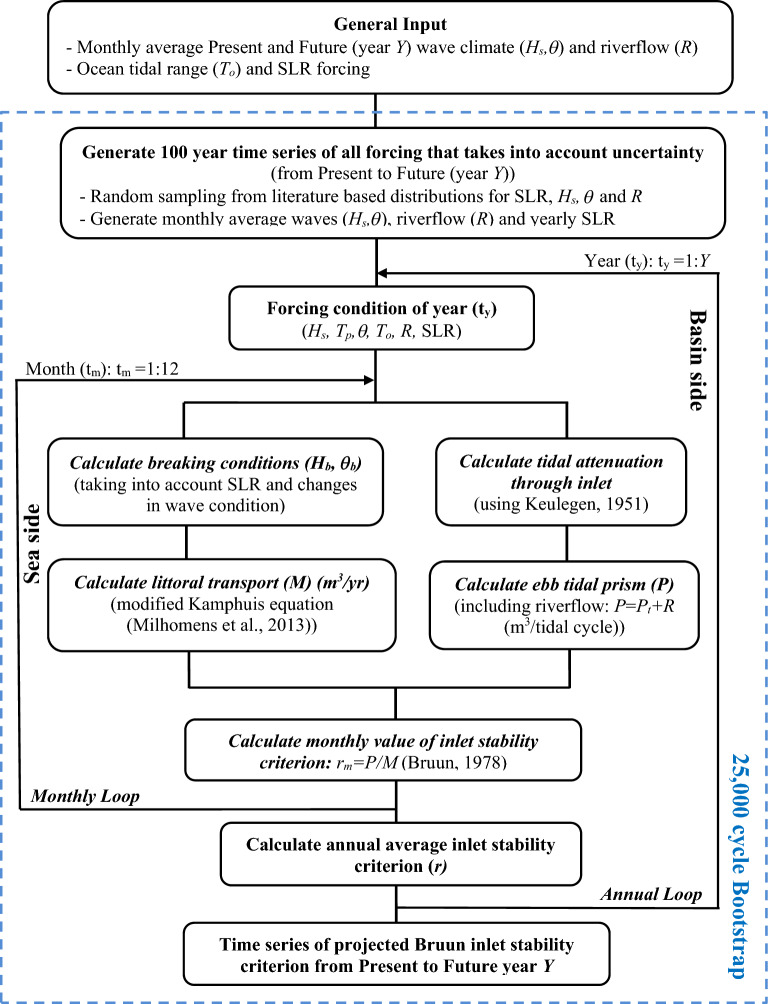
Basic structure of RAPSTA. *Y: *index of future year from present year, *t*_*y*_: year index in the range [1-*Y*] in the annual loop, *t*_*m*_: month index in the range [1–12] in the monthly loop, *H*_*s*_: significant wave height (m), *T*_*p*_: peak wave period (s), *θ*: wave direction (°), *H*_*b*_: breaking wave height (m), *θ*_*b*_: breaking wave angle (°), *R:* riverflow discharge (m^3^/s), *T*_*o*_: ocean tidal range (m), *SLR*: sea level rise at future year (m), *P*_*t*_: tidal prism (m^3^/tidal cycle), *P*: ebb tidal prism (m^3^/tidal cycle), *M*: longshore sediment transport (m^3^/year), *r*_*m*_: monthly inlet stability criterion, *r*: annual averaged inlet stability criterion.

The model developed and demonstrated here (RAPid assessment tool of inlet STAbilty – RAPSTA), calculates and tracks the temporal evolution of Bruun’s inlet stability criterion *r* (= P/M) using aggregated physical equations that represent the key processes governing inlet stability such as tidal attenuation, tidal prism and longshore sediment transport. The model takes into account climate change driven variations in mean sea level, wave characteristics and riverflows, and also the uncertainties in future projections of these system forcings. Using a monthly time-step, RAPSTA is capable of completing a 100 year simulation in under 3 seconds on a standard personal computer (PC). The basic structure of the model is summarized in Fig. 1.

The starting point of a RAPSTA application is the generation of required model forcing time series. For this type of reduced complexity long term modelling approach, it is sufficient to use monthly averaged forcing (unless such averaging results in large under-predictions of peak values, in which case a shorter averaging window may be required). In the demonstration applications here, historical data at the 3 case study STIs were used to generate the required monthly averaged time series of contemporary wave conditions and riverflow, while downscaled projections of climate change modified monthly averaged wave conditions and riverflow for the end-twenty-first century period were used to generate the end-century wave conditions and riverflow, also at monthly resolution. The global mean SLR projections given by Jackson and Jevrejeva^29^ were used to calculate an annually varying mean sea level (MSL) time series for the simulation duration. For simplicity, here it is assumed that all system forcing will linearly change from their present values to their projected end-century values. Cumulative distributions based on literature are used to account for the uncertainty in future forcing with respect to SLR, *H*_*s*_*, θ,* and *R*. For SLR, the distributions given by Jackson and Jevrejeva^29^ are used, while for the other 3 variables uniform distributions are used. Please see “Methods” section for more details of the model and its implementation at the 3 case study sites.

## Case study sites

Here RAPSTA was applied to one each of the 3 main STI Types listed above under a high-end greenhouse gas emissions scenario following RCP 8.5. All 3 systems are located along the south west coast of Sri Lanka, and are the same 3 STI systems at which the process based snap-shot modelling approach was previously applied by Duong et al.^13^, thus providing a means of comparing results obtained by the two different modelling approaches (process based snap-shot versus reduced complexity).

**Figure 2:**
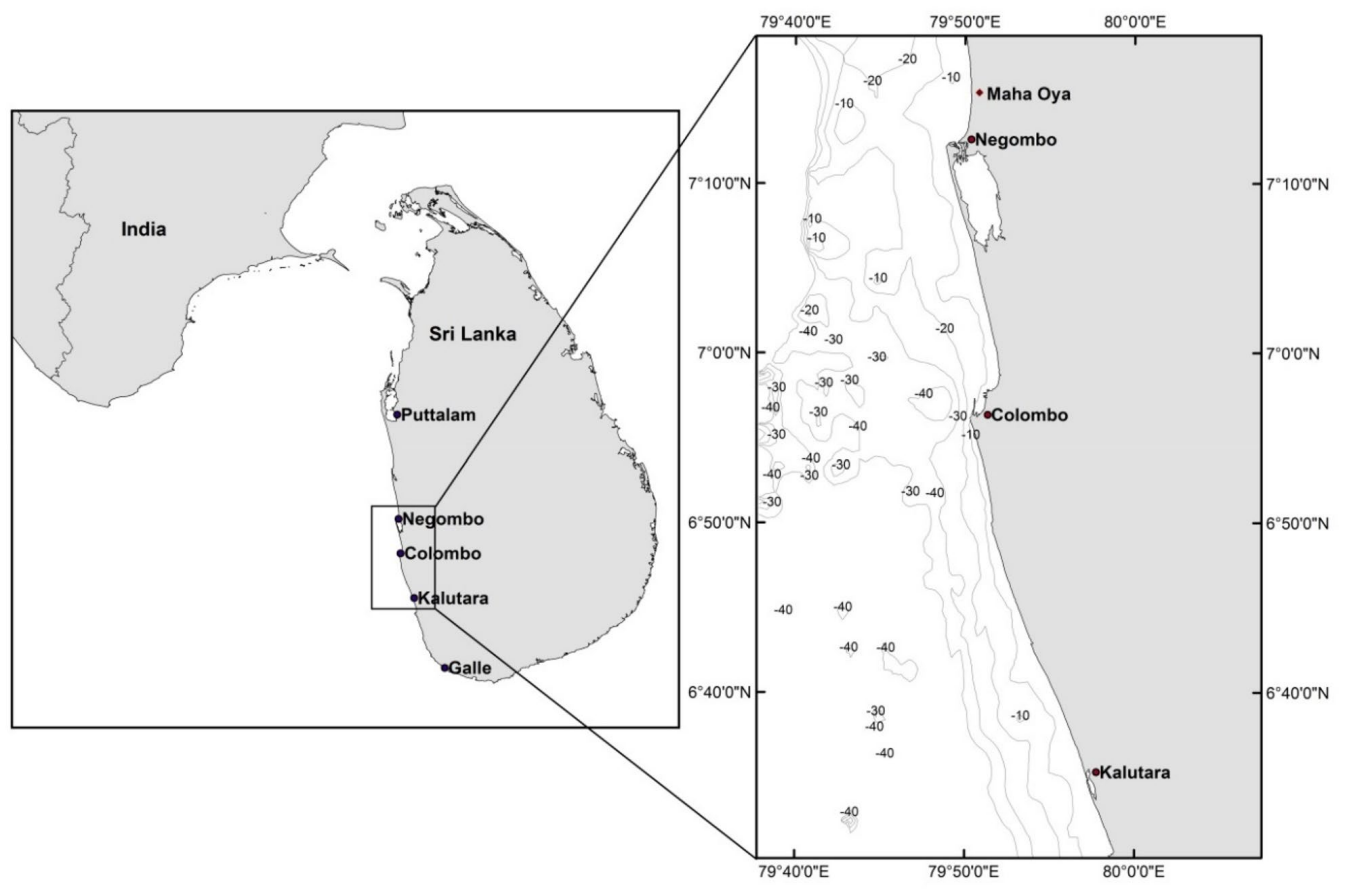
Location of Sri Lanka (left) and the 3 case study sites (right). The locations of the Capital city Colombo and other towns mentioned herein are also shown (Reproduced from Duong et al.^24^, all rights reserved).

Sri Lanka (pop: 22 million; area: 65,610 km^2^) is located southeast of India (Fig. 2) and experiences a tropical monsoon climate with 2 monsoon seasons; the Southwest (SW) monsoon from May to September and the Northeast (NE) monsoon from November to February. The annual rainfall in the country is highly seasonal with about one third of the total annual rainfall occurring between October and December^30^*.* The coastline of Sri Lanka is wave dominated (average offshore significant wave height of about 1.1 m) and micro-tidal (mean tidal range of about 0.5 m). The most energetic waves are experienced along the SW coast of Sri Lanka during the SW monsoon, with offshore significant wave heights ranging between 1 and 2 m. Most of the beaches along the coastline are composed of quartz sand with grain diameters (*D*_50_) varying between 0.2 and 0.45 mm^13^.

### Negombo lagoon (Type 1 STI)

**Figure 3:**
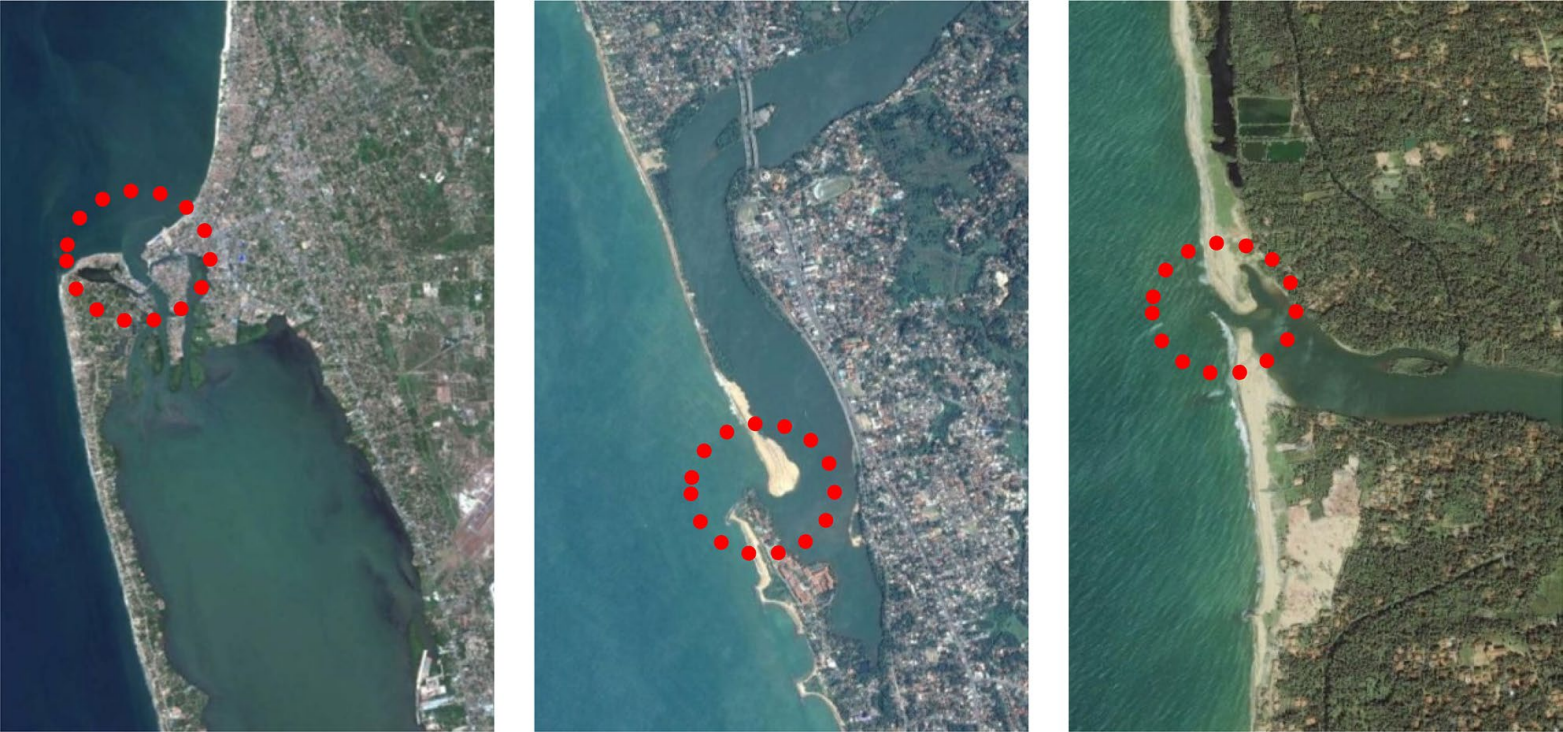
Negombo lagoon – Permanently open, locationally stable inlet: Type 1 (left), Kalutara lagoon – Permanently open, alongshore migrating inlet: Type 2 (middle), Maha Oya river – Seasonally/Intermittently open, locationally stable inlet: Type 3 (right). The red dotted circles indicate inlet location (Reproduced from Duong et al.^24^, all rights reserved).

Negombo lagoon, located about 30 km North of Colombo (Fig. 3, left), is connected to the ocean via a permanently open, relatively wide (400 m), short (300 m) and shallow (3 m) inlet which is locationally stable. The surface area and average depth of the lagoon are around 45 km^2^ and 1 m, respectively. Due to the sheltered nature of the inlet, the net annual longshore sediment transport rate in the vicinity of the inlet is insignificant^31^. The beaches adjacent to the inlet are sandy with a *D*_50_ of 0.25 mm^32^. Riverflow into the lagoon, which mostly occurs during the SW monsoon, is derived from Dandugam Oya, Ja Ela and several streams from Muthurajawela. The average annual riverflow (~ 90 m^3^/s) received by the lagoon varies from more than 200 m^3^/s during rainy seasons to almost nothing during dry seasons^32^.

### Kalutara lagoon (Type 2 STI)

Kalutara lagoon, located about 40 km South of Colombo (Fig. 3, middle), receives riverflow from the Kalu River, which is the second largest river in Sri Lanka in terms of annual riverflow volume ^13^. The Kalu inlet, in its natural state, is a permanently open, alongshore migrating inlet that is about 150 m wide, 150 m long and 4.5 m deep. Before the inlet was fixed in the south with breakwaters in the 1990s, the inlet has migrated alongshore following a 3–4 year cycle in which the inlet migrated around 2 km from north to south before a new, more hydraulically efficient inlet was either artificially or naturally incised at the northern end of the sand barrier^33^. The surface area of the Kalutara lagoon is about 2 km^2^ and the average depth in the lagoon is 3 m^24^. The net longshore sediment transport rate along the sand barrier is about 0.5 million m^3^/year to the south^13,34^. The sandy beaches near the inlet comprise of sand with a *D*_50_ of about 0.25 mm. The catchment of the Kalu river receives rainfall during both monsoons, resulting in river discharges that are higher than 100 m^3^/s most of the year (with peaks exceeding 280 m^3^/s in June and October). The average river discharge of the Kalu river is around 200 m^3^/s^13,35^.

### Maha Oya river (Type 3 STI)

Maha Oya inlet (Fig. 3, right), located about 40 km North of Colombo, is a narrow (100 m wide), short (70 m long), shallow (3 m depth) inlet which closes whenever the riverflow is small. The lagoon connected to the inlet has a surface area is about 0.2 km^2^ and an average depth of 3.5 m. The net longshore sediment transport near the inlet is about 0.5 million m^3^/year to the north^34^ (GTZ, 1994). The beach area adjacent to the inlet is sandy with a *D*_50_ of 0.25 mm. The Maha Oya river, with an average annual discharge of 1571 million m^3^/year, discharges into the ocean when the inlet is open. As the 1528 km^2^ catchment of Maha Oya derives most of its riverflow during the NE monsoon, peak discharges in the river (~ 140 m^3^/s) is usually in November, while the average discharge is about 50 m^3^/s^24^.

The key dimensions of the 3 systems, required as input to the model, are summarized in Table 2.

**Table 2:** Key dimensions for the 3 systems required as model input.

Inlet Type	Estuary/lagoon				Inlet			Beach
	Width (m)	Length (m)	Depth (m)	Basin area (km^2^)	Width (m)	Length (m)	Depth (m)	D_50_ (μm)
Type 1 (Negombo lagoon)	3500	13,000	1	45	400	300	3	250
Type 2 (Kalutara lagoon)	3500	500	3	1.75	150	150	4.5	250
Type 3 (Maha Oya river)	200	1000	3.5	0.2	100	70	3	250

## Model forcing

### Baseline conditions (year 2000)

**Figure 4:**
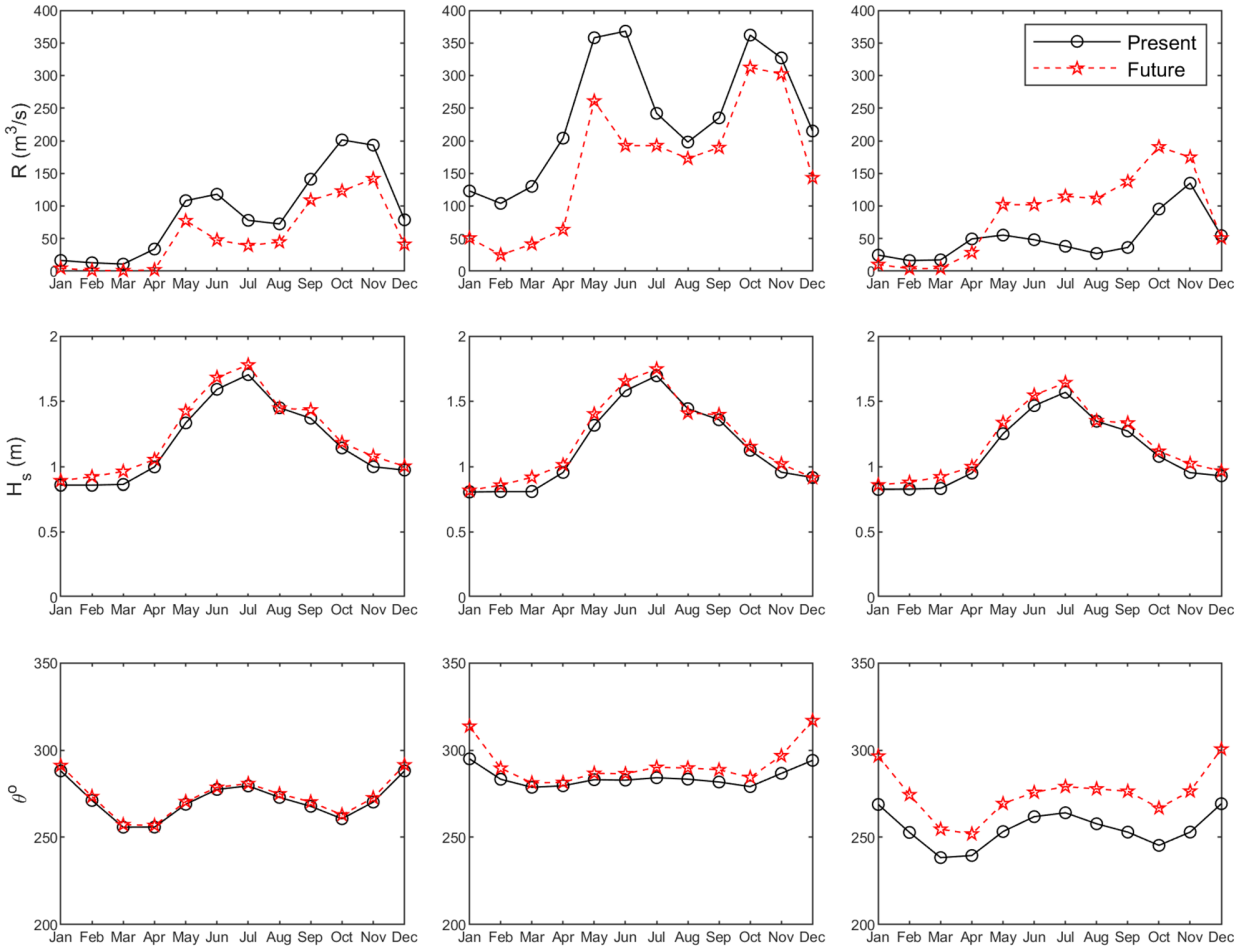
Contemporary and projected end-century forcing time series for Negombo lagoon (left), Kalutara lagoon (middle), and Maha Oya river (right); Top: riverflow (*R*), Middle: Significant wave height (*H*_*s*_) at 10 m depth, Bottom: Mean wave direction (*θ*) at 10 m depth.

Based on published values^32,36^, the mean ocean tidal range for all 3 systems (which are located along a single coastal stretch spanning about 100 km) was taken as 0.5 m. Time series of monthly average riverflow were constructed for the 3 systems (Fig. 4, top) based on available sparse data and local publications^24^. Wave conditions at 10 m depth for each case study site (Fig. 4, middle and bottom) were extracted from a regional SWAN model extending from Galle to Puttalam along the SW coast of Sri Lanka, forced with measured offshore wave conditions^13,24^.

### End-twenty-first century conditions

The end-century riverflow and wave conditions used in this study were derived from Duong et al.^13^, which are based on regional scale hydrological Catchment Land Surface Model (CLSM) and wave (SWAN) models driven by dynamically downscaled future climate conditions. For SLR, distributions of global mean SLR projections for RCP 8.5 presented by Jackson and Jevrejeva^29^ were used for all 3 systems (see “Methods” section for more details).

The baseline (2000) and end-century riverflow and wave conditions used in this study are shown in Fig. 4. With the annual forcing shown in Fig. 4 as the start and end conditions, RAPSTA was applied over the twenty-first century to each case study site, while sampling SLR, *H*_s_, *θ,* and *R* from fitted distributions to capture the uncertainty associated with future forcing (see “Methods” section).

## Model projections of inlet stability

**Figure 5:**
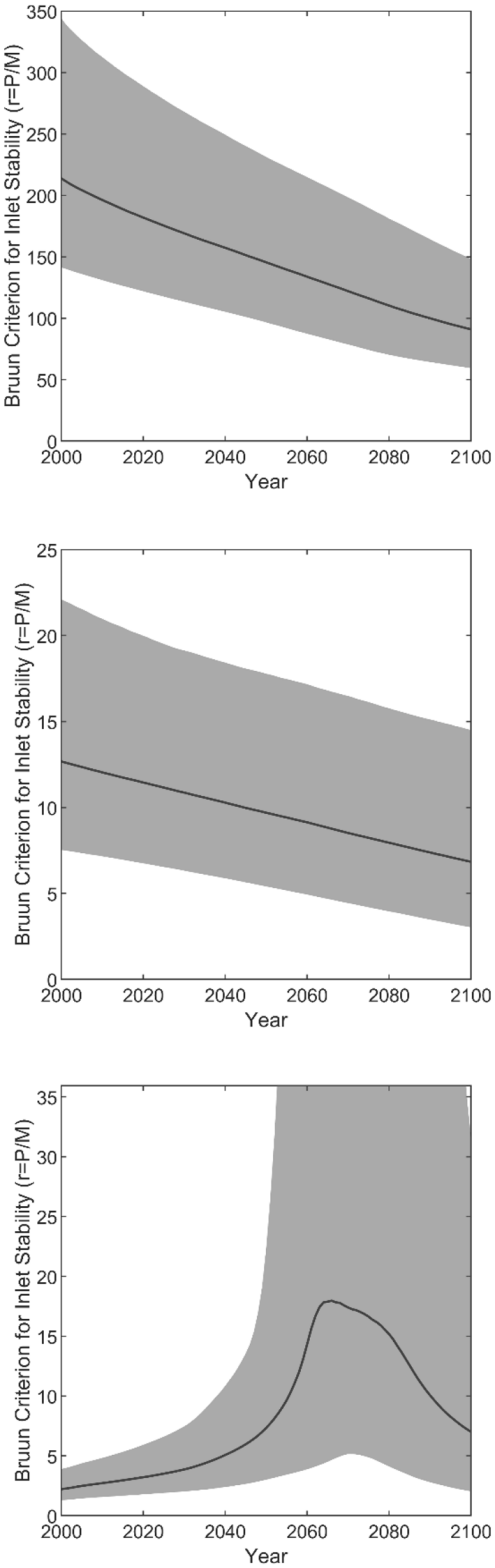
RAPSTA model results for Negombo lagoon (top), Kalutara lagoon (middle), and Maha Oya river (bottom). The solid line shows the median projection and shaded area shows the 95% confidence limits.

Model predicted temporal variations of inlet stability for the 3 systems are shown in Fig. 5 (time series of *P* and *M* (median and 95% uncertainty range) generated for the 3 case studies using the approaches described above are shown in Fig. S1).

First, we compare the end-century *r* values and inlet Type projected by the model with those projected for the same time horizon by Duong et al.^13^ using the process based snap-shot approach (Table 3).

**Table 3:** Comparison of end-century, RCP 8.5 median *r* values and inlet Type projected by the reduced complexity model RAPSTA with the projections obtained from the process based snap-shot approach of Duong et al. ^13^ for the 3 STIs.

Model	*r* (2100)	Trend of *r* change from present	Bruun classification	Inlet Type
**Negombo lagoon (Present condition: Type 1 – Permanently open, locationally stable, ***r*** = 221)**				
RAPSTA	90	Decrease	Stable (fair to poor)	Type 1
Process based snap-shot model	75	Decrease	Stable (fair to poor)	Type 1
**Kalutara lagoon (Present condition: Type 2 – Permanently open, alongshore migrating, ***r*** = 11)**				
RAPSTA	7	Decrease	Unstable	Type 2 or 3
Process based snap-shot model	6	Decrease	Unstable	Type 2
**Maha Oya river (Present condition: Type 3 – Seasonally/intermittently open, locationally stable, ***r*** = 1)**				
RAPSTA	7	Increase	Unstable	Type 2 or 3
Process based snap-shot model	5	Increase	Unstable	Type 3

The present conditions are from Duong et al.^13^.

Table 3 shows that, for all 3 systems, the end-century projections for median *r* and inlet Type given by RAPSTA are in agreement with those projected with the process based snap-shot modelling approach presented by Duong et al.^13^, providing confidence in both approaches.

Next, we summarise the time evolution of inlet stability projected by RAPSTA (based on median values only) for the 3 case studies and compare these findings with those presented by Duong et al.^13^ using the process based snap-shot modelling approach (see Figs. S2–S4 for Duong et al.’s^13^ process based snap-shot model results). The implications of considering the projection uncertainties are addressed in the “Discussion” section.

### Negombo lagoon (Type 1)

The model predicts that the level of stability (i.e*. r* value) of this system will gradually decrease in time (Fig. 5, top). This is because while *P* increases by about 50% from the present to 2100, *M* increases by almost 150% (Fig. S1, top). Table 3 indicates that Negombo lagoon will remain a locationally and cross sectionally stable Type 1 inlet by 2100, which is in agreement with the corresponding process based snap-shot model predictions presented by Duong et al.^13^ (see also Fig. S2). However, the level of stability decrease shown in Fig. 5 (top) indicates that the inlet will deteriorate from a highly stable one (*r* > 150 at present) to an inlet with fair to poor stability (50 < *r* < 100) according the Bruun criteria for inlet stability (Table 1).

### Kalutara lagoon (Type 2)

Model predictions for 2100 are comparable to those of the corresponding process based snap-shot model predictions^13^ in terms of all diagnostics considered in Table 3. In this case too, the model predicts that the level of stability will decrease over time (Fig. 5, middle). This is due to the 60% increase in *M* while *P* only decreases by about 15% during the 100 year simulation (Fig. S1, middle). While the computed reduction of *r* from its present value of 11 to 7 by 2100 is quantitatively small, this moves the inlet stability into the uncertain 5 < *r* < 10 range after around 2045, where the inlet could be Type 2 or Type 3 as indicated in Table 1. Therefore, the only conclusion that can be drawn from the reduced complexity model, which completely depends on the *r* vs inlet Type relationship suggested in Table 1 for projecting inlet stability, is that the inlet could be either a Type 2 or Type 3 inlet by the end of the twenty-first century. On the other hand, Duong et al.’s^13^ process based snap-shot model result for the end of the twenty-first century at Kalutara inlet, while giving an *r* value of 6 (also in 5 < *r* < 10 range), provides a more definitive projection that this STI will remain as an alongshore migrating Type 2 inlet, based on the 2-dimensional morphodynamic evolution simulated in that study (see Fig. S3). This highlights that when 5 < *r* < 10, the reduced complexity model should ideally be used in conjunction with the process based snap-shot modelling approach to obtain a confident result.

### Maha Oya river (Type 3)

In this case too, end-century RAPSTA projections are comparable to those of the corresponding process based snap-shot model predictions^13^ in terms of the diagnostics considered in Table 3. Similar to the Kalutara lagoon inlet projections, here too the end-century *r* projection falls in the uncertain 5 < *r* < 10 range where the inlet could be Type 2 or Type 3, whereas Duong et al.’s ^13^ process based modelling approach projects a Type 2/3 borderline *r* value of 5 (see also Fig. S4). However, due to its ability to provide the full temporal evolution of *r* over the 100 year study period, RAPSTA provides very interesting insights that the process based snap-shot modelling approach does not provide. Figure 5 (bottom) shows that median *r*, which remains below 10 till about 2055, rapidly increases to reach a maximum of about 18 between 2065 and 2075, and then drops back to below 10 soon after 2090. This is because, while *P* keeps steadily increasing through the 100 year simulation, *M* (median) goes through a zero crossing around 2070 and changes direction from a northward transport to a southward transport due to the projected climate change induced clockwise rotation of wave direction in the study area (Fig. S1, bottom). Due to this phenomenon, when northward *M* decreases towards zero from about 2055, *r* increases rapidly, and then drops again when southward *M* starts to become substantial after 2075. This implies that Maha Oya inlet may turn into a permanently open, alongshore migrating Type 2 inlet during 2055–2090.

## Discussion

### Model uncertainties versus forcing uncertainties

All numerical models reduce system physics into a set of solvable mathematical expressions. Reduced complexity models, such as RAPSTA, reduce system physics to a very high degree in order to achieve the speed that is necessary to perform the thousands of simulations required to quantify the forcing uncertainty that is inherent in any climate impact study. Therefore, it should be borne in mind that, while the model applications presented here quantify the forcing uncertainty, they do not quantify model uncertainty. This could, for example be done by considering ranges of values (within reasonable bounds) for the various model parameters used in the model and decomposing the uncertainty in the projections to model versus forcing uncertainties using a Sobol indices approach as done by Le Cozannet et al.^37^ and Athanasiou et al.^38^. Such an analysis however falls outside the scope of this study.

### Implications of projection uncertainties

The above discussion of model results was limited to median projections. Inspection of the 95% confidence ranges produced by RAPSTA provides further insights that cannot be obtained from deterministic model applications such as that presented by Duong et al.^13^. Figure 5 shows that the *r* value of the Negombo lagoon inlet stays above 50 throughout the twenty-first century even when the projection uncertainties are taken into account, indicating that there is a high level of confidence that this system will remain a Type 1 inlet at least till the end of the century. However, this is not the case for the other two STIs considered in this study. For example, while the upper limit of the 95% confidence range of *r* for the presently Type 2 Kalutara lagoon inlet stays in the 10–20 range throughout the twenty-first century, the lower limit drops below 5 around 2060. This indicates that while there is a chance that this presently Type 2 system may remain a Type 2 STI until the end of the century, there is also a chance that it becomes a Type 3 inlet from 2060 onwards. At the presently Type 3 Maha Oya inlet, while the lower limit of the 95% confidence range of *r* stays below 5 throughout the twenty-first century, the upper limit increases above 10 around 2040, further increasing beyond 20 around 2050. This indicates that Maha Oya inlet could either remain as a Type 3 STI throughout the century, or become a Type 2 inlet in about 20 years from now, and change further into a stable Type 1 inlet by mid-century and remain as such until the end of the century. These uncertainties highlight the need for future adaptation strategies to remain flexible.

### Limits of RAPSTA applicability

RAPSTA is specifically designed for STIs, as defined by Duong et al.^6^. As such, the model does not take into account complex processes such as ebb shoal or sand bar attachments to the shoreline, episodic inlet breaching, channel bifurcation, effects of extreme events etc. The model is designed to simulate the “mean” long term inlet stability and does not represent short term responses, which may nevertheless, have some impact on long-term inlet stability.

## Conclusions

Climate change driven variations in mean sea level, wave conditions and riverflows are expected to affect the stability of the thousands of small tidal inlets (STIs) spread around the world. To inform the development of effective adaptation strategies for the coastal areas in the vicinity of these inlets, it is necessary to know how inlet stability will change over time, and if/when such changes in inlet stability might result in a regime change, shifting them from one inlet Type to another. However, numerical models and modelling approaches available to date are unable to perform continuous morphodynamic simulations over climate change time scales (50–100 years), and certainly do not lend themselves to probabilistic applications, requiring thousands of individual simulations, that are needed to quantify the forcing uncertainty associated with climate projections. To address this need, here we developed and piloted a probabilistic, reduced complexity model (RAPSTA – RAPid assessment tool of inlet STAbility) to obtain rapid assessments of the temporal evolution of the stability of STIs. The model is very easy to use and provides a 100 year projection in less than 3 seconds on a standard PC.

Based on aggregated physical equations, RAPSTA calculates and tracks the temporal evolution of Bruun’s inlet stability criterion *r* (= *P/M*, where *P* = tidal prism (m^3^) and *M* = annual longshore transport (m^3^/year)). The main physical processes considered in RAPSTA are tidal attenuation, tidal prism and longshore sediment transport. The model accounts for climate change driven variations in mean sea level, wave characteristics and riverflows, while preserving seasonal signals in these forcings. The model is demonstrated here at 3 STIs representing the 3 main Types of STIs present around the world; Negombo lagoon – Permanently open, locationally stable inlet (Type 1); Kalutara lagoon – Permanently open, alongshore migrating inlet (Type 2); and Maha Oya inlet – Seasonally/Intermittently open, locationally stable inlet (Type 3). The 3 case study sites are located along an approximately 100 km stretch of coastline along the south west coast of Sri Lanka.

End-century projections of inlet stability provided by RAPSTA for the 3 case study sites, under a high greenhouse gas emissions scenario following RCP 8.5, are comparable to those obtained using a process based snap-shot modelling approach by Duong et al.^13^ for the same sites. RAPSTA projections for the 3 sites indicate that:

- The Negombo lagoon inlet will remain as a Type 1 inlet during the twenty-first century. The level of inlet stability will deteriorate from *highly stable* (*r* > 150 at present) to *fair to poor* (50 < *r* < 100) at the worst, even when projection uncertainties are taken into account.
- The median *r* value for presently Type 2 Kalutara lagoon inlet will decrease into the Type 2/3 range of 5 < *r* < 10 around 2045 and will remain in that range until the end of the century. While Table 1 indicates that, in this range of *r*, the inlet could be either a Type 2 or Type 3 inlet, the corresponding process based snap-shot model results show that Kalutara lagoon will be a Type 2 inlet by the end of the twenty-first century. Consideration of the 95% confidence range of the projections indicate that while there is a chance that this system may remain a Type 2 STI until the end of the century, there is also a chance that it becomes a Type 3 inlet from 2060 onwards.
- By the year 2100, the median *r* value of Maha Oya inlet falls in the Type 2/3 range of 5 < *r* < 10, indicating that this presently Type 3 system could be either be a Type 2 or Type 3 inlet by the end of the century, which is consistent with the projections derived from the process based snap-shot modelling approach. Due to its ability to provide the full temporal evolution of *r* over the 100 year study period, RAPSTA median *r* projections indicate that this system may turn into a Type 2 inlet during 2055–2090. However, the temporal evolution of the 95% confidence range of *r* for the Maha Oya inlet indicates that there is a chance that this system could either remain as a Type 3 STI throughout the century or become a Type 2 inlet around 2040, turning into a stable Type 1 inlet for the entire second half of the century.

## Methods

### Climate change forcing

#### Downscaling of climate variables

The same dynamically downscaled climate variables used by Duong el al.^13^, which were derived from the stretched grid model CCAM (Conformal Cubic Atmospheric Model)^39^, were used in this study. In Duong et al.^13^, CCAM was forced with Sea Surface Temperatures from ECHAM and GFDL (CMIP 3 generation), two of the GCMs that performed well in the study area. Six hourly CCAM outputs including winds, surface temperature, atmospheric pressure, radiation, ocean temperature etc. were obtained for the 1981–2000 (baseline) and 2081–2100 (end-century) time slices at a grid resolution of about 50 km over Sri Lanka.

### Climate change and riverflow

The riverflow projections used here are the same projections used by Duong et al.^13^. These projections were derived for the present (1981–2000) and future (2081–2100)^40^ by applying dynamically downscaled projections of climate variables from CCAM^39^ in the Catchment Land Surface Model (CLSM^41,42^). Mahanama and Zubair^40^ presented bias corrected (using gridded precipitation data), downscaled ECHAM and GFDL precipitation hindcasts for both time slices. These projections indicate that riverflow will decrease by about 41% and 32% at Negombo lagoon and Kalutara lagoon respectively, and increase by about 72% at Maha Oya river. The different direction of change in riverflow projected for Maha Oya is because most of its catchment is located in the dry zone, which receives most of its rainfall during the weaker NE monsoon, as opposed to the catchments of other two study sites that are mostly located in the wet zone receiving most of its rainfall during the stronger SW monsoon. Climate model projections indicate that the dry zone of Sri Lanka will become wetter while its wet zone will become drier^40^.

### Climate change and waves

Here too, the same dynamically downscaled 6-houly offshore wave conditions used by Duong et al.^13^ were used. These wave projections were obtained by Bamuanawala et al.^43^ using CCAM winds to force two nested spectral wave models WAVEWATCH III (for far field) and SWAN (for near field) for 1981–2000 (hindcast) and 2081–2100 (future) time slices. Future projections were bias corrected using wave measurements at Colombo^43^.

### Sea level rise

For sea level rise, here we used the probabilisitic global mean sea level rise projections given by Jackson and Jevrejeva^29^. These projections combine the major drivers of SLR including steric sea-level change, dynamic sea-level change, surface mass balance and ice dynamics of the Greenland and Antarctic ice sheets, surface mass balance of ice from glaciers and ice-caps, and Glacial Isostatic Adjustment. It should be noted that local processes such as land subsidence are not included in these SLR projections.

### Climate change and longshore sediment transport

At the most basic level, longshore sediment transport (LST) rate is a function of sediment size (*D*_*50*_) and breaking wave height (*H*_*b*_) and angle (*θ*_*b*_). There are several bulk equations (e.g. CERC^44^; Bayram et al.^45^) that express LST as a predominant function of these parameters. Kamphuis^46^ presented an LST equation which, apart from these parameters, also includes the effect of the surf zone bed slope (Eq. (1)).

1 $${I}_{m}=2.27{H}_{b}^{2}{T}_{p}^{1.5}{{m}_{b}}^{0.75}{D}_{50}^{-0.25}{\mathrm{sin}}^{0.6}\left(2{\theta }_{b}\right)$$

where, ${I}_{m}$ is immersed mass of sediment transported alongshore (kg/s), ${H}_{b}$ is the significant wave height at the breaker (m), ${\theta }_{b}$ is the wave angle at the break point (degrees), ${T}_{p}$ is the peak wave period (s), ${m}_{b}={h}_{br}/{\lambda }_{br}$ is the beach slope, ${h}_{br}$ is the water depth at break point (m), ${\lambda }_{br}$ is the distance from the shoreline to the break point (m), and ${D}_{50}$ is the median grain size (m).

${I}_{m}$ is related to the volume via ${Q}_{l}=\frac{{I}_{m}}{\left({\rho }_{s}-\rho \right)\left(1-p\right)}$ , where ${Q}_{l}$ is the sediment transport volume (m^3^/s), ${\rho }_{s}$ is the density of sand (kg/m^3^), $\rho$ is the fluid density (kg/m^3^), $p$ is porosity of sand.

This equation has been shown to perform remarkably well on large data sets^46–48^. Mil-Homens et al.^48^ re-assessed the commonly used CERC, Bayram and Kamphuis LST equations using a comprehensive database containing laboratory and field data and proposed new improved versions for all 3 equations. Among these however, the improved Kamphuis equation (Eq. (2)) gave the best agreement with data.

2 $${I}_{m,new}=0.149{H}_{b}^{2.75}{T}_{p}^{0.89}{{m}_{b}}^{0.86}{D}_{50}^{-0.69}{\mathrm{sin}}^{0.5}\left(2{\theta }_{b}\right)$$

Climate change will result in sea level rise and modified deepwater wave heights and directions around the world^49,50^. Such climate change driven modifications in offshore wave conditions will also affect breaking wave properties *H*_*b*_ and *θ*_*b*_. Furthermore, SLR will result in waves breaking closer to the shoreline and hence a steeper surf zone slope (higher *m*_*b*_). All of these climate change effects can be accounted for by using Eq. (2) with appropriate climate change forcing.

Breaking wave conditions, required by Eq. (2) to calculate LST, were not directly extracted from the regional SWAN wave models used here as model resolution (500 m) is insufficient to adequately resolve surf zone dynamics. Instead wave conditions extracted from the regional SWAN model at 10 m depth were transformed to the break-point using Snell’s law for wave refraction and the wave dispersion relation (Eq. (3)), assuming that nearshore depth contours are parallel to the coastline and follow a Dean equilibrium profile corresponding to the measured *D*_50_ of the study area. This provides *H*_*b*_, *θ*_*b*_ and the depth at which waves break (*h*_*br*_). The breaker depth (*h*_*br*_) is then used together with the concurrent MSL to calculate surf zone slope *m*_*b*_ required by Eq. (2). Subsequently, the monthly time series of *H*_*b*_, *θ*_*b*_ and *m*_*b*_ were used in Eq. (2) to obtain monthly LST values for the entire simulation.

3 $${\omega }^{2}=gk\mathrm{tanh}\left(kh\right)$$

where, $\omega =\frac{2\pi }{T}$ is the angular frequency (rad/s), $k=\frac{2\pi }{\lambda }$ is the wave number (rad/m), λ is the wave length (m), and *h* is the water depth (m).

### Climate change and tidal prism

The total ebb tidal prism can be divided into two parts:

4 $$P={P}_{t}+R$$

where, *P*_*t*_ is the ebb flow due to tide only and *R i*s the riverflow volume during ebb. In small estuary/lagoon system where it can safely be assumed that there is no phase lag in tidal elevation within the system^51^, *P*_*t*_ can be expressed as:

5 $${P}_{t}={A}_{b}\times \left(2\times {a}_{b}\right)$$

where, *A*_*b*_ is the surface area of the estuary/lagoon and *a*_*b*_ is the mean tidal amplitude inside the estuary/lagoon.

As STIs do not usually contain extensive tidal flats or salt marshes^10,13,24^, it can be assumed that SLR will not change *A*_*b*_. The lagoon tidal range *a*_*b*_ then is a function of the ocean tidal range and tidal attenuation across the inlet channel^51–53^. Climate change is not expected to affect ocean tides in a significant way and here it is assumed that future *a*_*b*_ can only be affected by changes in tidal attenuation. Inlet channel dimensions such as length, width and depth will directly affect the degree of tidal attenuation^51^. With climate change, the competing effects of SLR and basin infilling^8–10^ will result in increasing inlet depth at STIs by half the amount of SLR as shown by Ranasinghe et al.^10^. This will decrease tidal attenuation, thus increasing *P*_*t*_*.*

Keulegan^51^ presented an analytical solution to calculate tidal attenuation at inlets which can be used to calculate the climate change modified *P*_*t*_ at STIs. The starting point for this approach is Eq. (6) which describes the difference in water level across a tidal inlet:

6 $$\frac{{dh}_{b}}{dt}=K\sqrt{{h}_{o}-{h}_{b}}$$

where, ${h}_{b}$ is the water level inside the basin (m), ${h}_{o}$ is the water level at the sea (m), $K$ is the coefficient of filling or repletion, given by the Eq. (7) below:

7 $$K=\frac{{T}_{t}}{2\pi {a}_{o}}\frac{A}{{A}_{b}}\sqrt{\frac{2gr{a}_{o}}{fL+mr}}$$

where, ${T}_{t}$ is the tidal period (s), ${a}_{o}$ is the tidal amplitude at the sea (m), $A$ is the cross-sectional area of the connecting channel (m^2^), ${A}_{b}$ is the basin surface area (m^2^), $g$ is the gravitational acceleration (m^2^/s), $r$ is the hydraulic radius of the channel (m), $f$ is the friction coefficient, $L$ is the length of the connecting channel (m), $m$ is the coefficient resulting from the velocity distribution over the channel cross section.

As Eq. (6) is an implicit equation, after some mathematical manipulations and approximations for sinusoidal tidal oscillations, Keulegan^51^ arrived at Eq. (8) which is explicit.

8 $$\frac{{a}_{b}}{{a}_{o}}=\sin\tau$$

where, ${a}_{b}$ is the tidal amplitude inside the basin (m), and sinτ is a function of *K*.

Keulegan^51^ further provided look-up tables for sinτ vs *K*. As the latter can easily be estimated for a given system from Eq. (7), *a*_*b*_ can then be calculated for a given ocean tidal amplitude *a*_*o*_, which is generally known. This *a*_*b*_ can then be used, along with *A*_*b*_, in Eq. (5) to compute *P*_*t*_*,* which can be combined with a known riverflow (per ebb phase) to calculate *P* using Eq. (4). It is noted that Keulegan’s^51^ approach does not allow *a*_*b*_ to be greater than *a*_*o*_ (i.e. $\sin\tau$ always < 1) and therefore is not suitable for situations where tidal resonance may amplify *a*_*b*_*.* However, for tidal resonance to occur the ratio length of basin/tidal wave length should be 0.25^54^. For STIs therefore this situation is unlikely to occur. For example, the length of basin/tidal wave length ratio of the 3 STIs investigated here ranges between 0.002 and 0.078.

To calculate the monthly averaged time series of ebb tidal prism *P*, first the time series of *P*_*t*_* i*s required. In the model developed here, this is achieved by using Eqs. (4)–(6) above. The time variation of *P*_*t*_ arises from the time variation of the repletion coefficient *K* (Eq. (7)) which varies due to the SLR/basin infilling driven increase in inlet depth (relative to MSL). As SLR is only updated annually, *P*_*t*_ will only change every year. The riverflow *R* (per ebb phase) time series, which varies monthly, is then added to the *P*_*t*_ time series to produce monthly *P* values for the model duration.

### Climate change and Bruun inlet stability criterion

Next, using the time series of *P* and LST values thus obtained, a monthly time series of the Bruun criterion *r*_*m*_ is generated using:

9 $${r}_{m}=\frac{P}{M}$$

Finally, the annual representative *r* value is obtained by taking the average of *r*_*m*_ over every year of the simulation.

### Probabilistic framing

As described above, LST is a function of, amongst other properties, wave conditions (height, period and direction) at the breaking point, which further depends on large scale bathymetry and SLR. Under the assumptions invoked for STIs here, the tidal prism will vary with future variations in riverflow and SLR/basin infilling. Hence, there are four key uncertain parameters to consider in RAPSTA: SLR, wave height, wave direction and riverflow.

To account for the uncertainty in SLR, here we directly sample SLR from the distributions given for RCP 8.5 by Jackson and Jevrejeva^29^. The uncertainty in wave conditions are accounted for by using a ± 42% uncertainty in the change of wave height (Eq. (10)) and a ± 25% uncertainty in the change of wave direction (Eq. (11)), following the projections for the study area presented by Semedo et al.^55^ and Lemos et al.^56^. In both cases, uniform distributions were used.

For significant wave height (*H*_*s*_), this was achieved by:

10 $${H_{s,BS}} (Y) = {H_{s}} (2000) + \{ {H_{s}} (2100) - {H_{s}} (2000)\} \times \frac{Y - 2000}{{100}} \times {\text{Uniform}}\left[ { - \,0.42,\,0.42} \right]$$

where, $H_{s,BS}$ is the bootstrapped significant wave height in a particular simulation, Uniform [-] is a uniformly distributed random number between − 0.42 and 0.42 (one value for each bootstrapped simulation) and *Y* is the year of the simulation (present is *Y* = 2000 and future is *Y* = 2100).

Similarly, the uncertainty in *θ* is accounted for by*:*

11 $${\theta_{BS}} (Y) = \theta (2000) + \{ \theta (2100) - \theta (2000)\} \times \frac{Y - 2000}{{100}} \times {\text{Uniform}}\left[ { - \,0.25,\,0.25} \right]$$

where, $\theta_{BS}$ is the bootstrapped wave direction.

As the uncertainty in riverflow projections is very high, the riverflow uncertainty was here accounted for by allowing a factor 2 deviation from projected riverflows and using:

12 $$R_{BS} (Y) = R(2000) + \{ R(2100) \times {\text{Uniform}}\left[ {0.5,2} \right] - R(2000)\} \times \frac{Y - 2000}{{100}}$$

where, *R*_*BS*_ is the bootstrapped riverflow.

For each monthly time step of the RAPSTA simulation, these 4 uncertain parameters were sampled using the above distributions within a Monte Carlo framework. The 100-year RAPSTA simulations were bootstrapped to obtain statistically stable projections, with convergence being achieved for both the median and 95% confidence range with a 25,000 cycle bootstrapping.

## Supplementary Information

Supplementary Figures.

## Data Availability

The data supporting the calculation and conclusions presented in this manuscript will be made available by the corresponding author, without reservation, to any qualified researcher.
