# Supplementary material for: Probabilistic projections of the stability of small tidal inlets at century time scale using a reduced complexity approach
Source: Sci Rep. 2021 Nov 25;11:22921. doi: 10.1038/s41598-021-01945-5 (PMC8617055; doi:10.1038/s41598-021-01945-5)
Supplement: Supplementary file 1 — Supplementary Figures. [file 41598_2021_1945_MOESM1_ESM.pdf]

Supplementary Information

PROBABILISTIC PROJECTIONS OF THE STABILITY OF SMALL TIDAL INLETS AT CENTURY TIME SCALE USING A REDUCED COMPLEXITY APPROACH

Trang Minh Duong[1,2,3,], Roshanka Ranasinghe[1,2,3], David P. Callaghan[4]*

[1] Department of Coastal & Urban Risk & Resilience, IHE Delft Institute for Water Education, P.O. Box 3015, 2601 DA, Delft, The Netherlands

[2] Department of Water Engineering & Management, University of Twente, P.O. Box 217, 7500 AE, Enschede, The Netherlands

[3] Harbour, Coastal and Offshore Engineering, Deltares, P.O. Box 177, 2600 MH, Delft, The Netherlands

[4] School of Civil Engineering, The University of Queensland, Brisbane, QLD, 4072, Australia

** corresponding author (t.duong@un-ihe.org)*

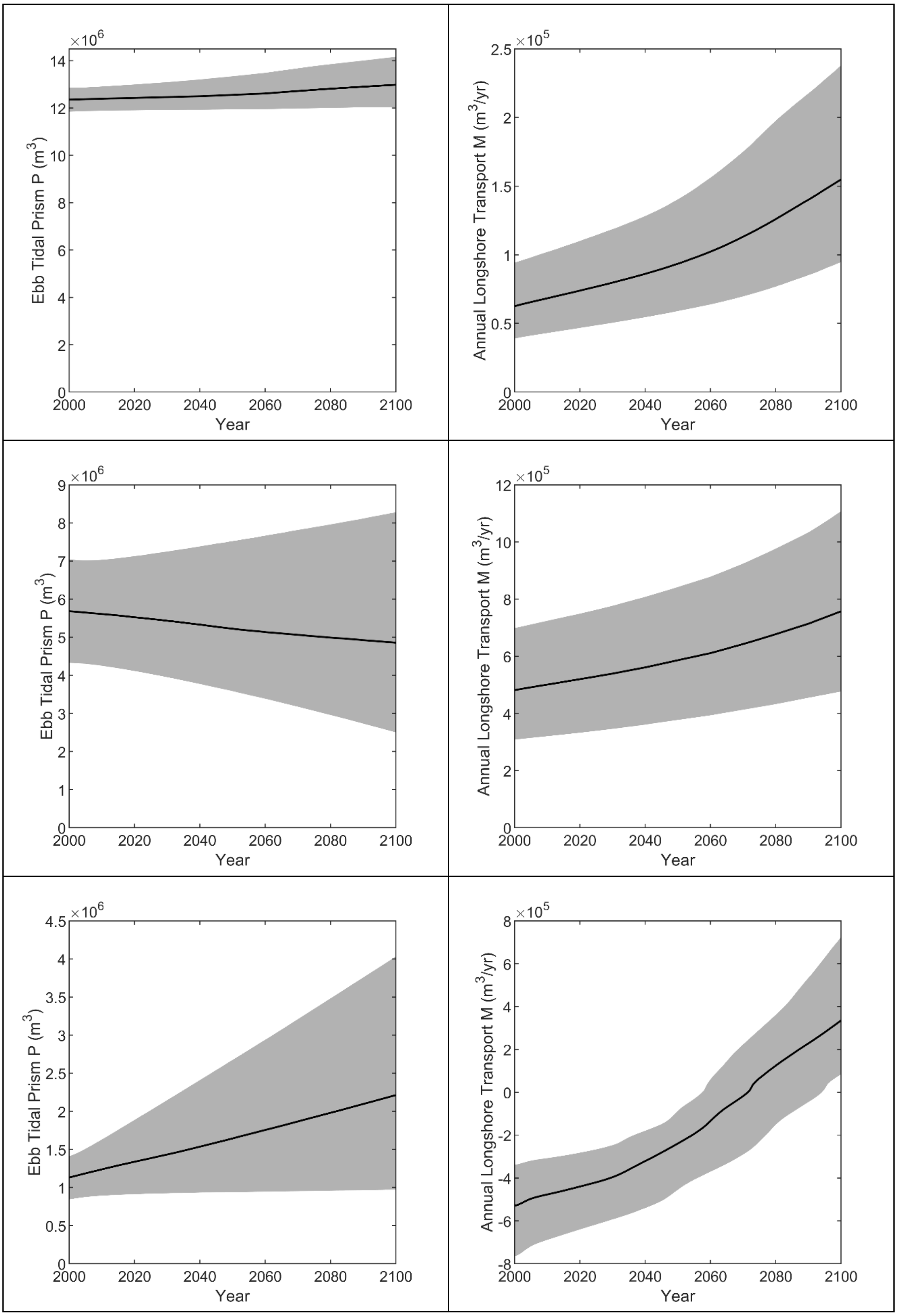

Figure S1. Time evolution of P (left) and M (right) for Negombo lagoon (top), Kalutara lagoon (middle), and Maha Oya river (bottom). M+ indicates southwards transport.

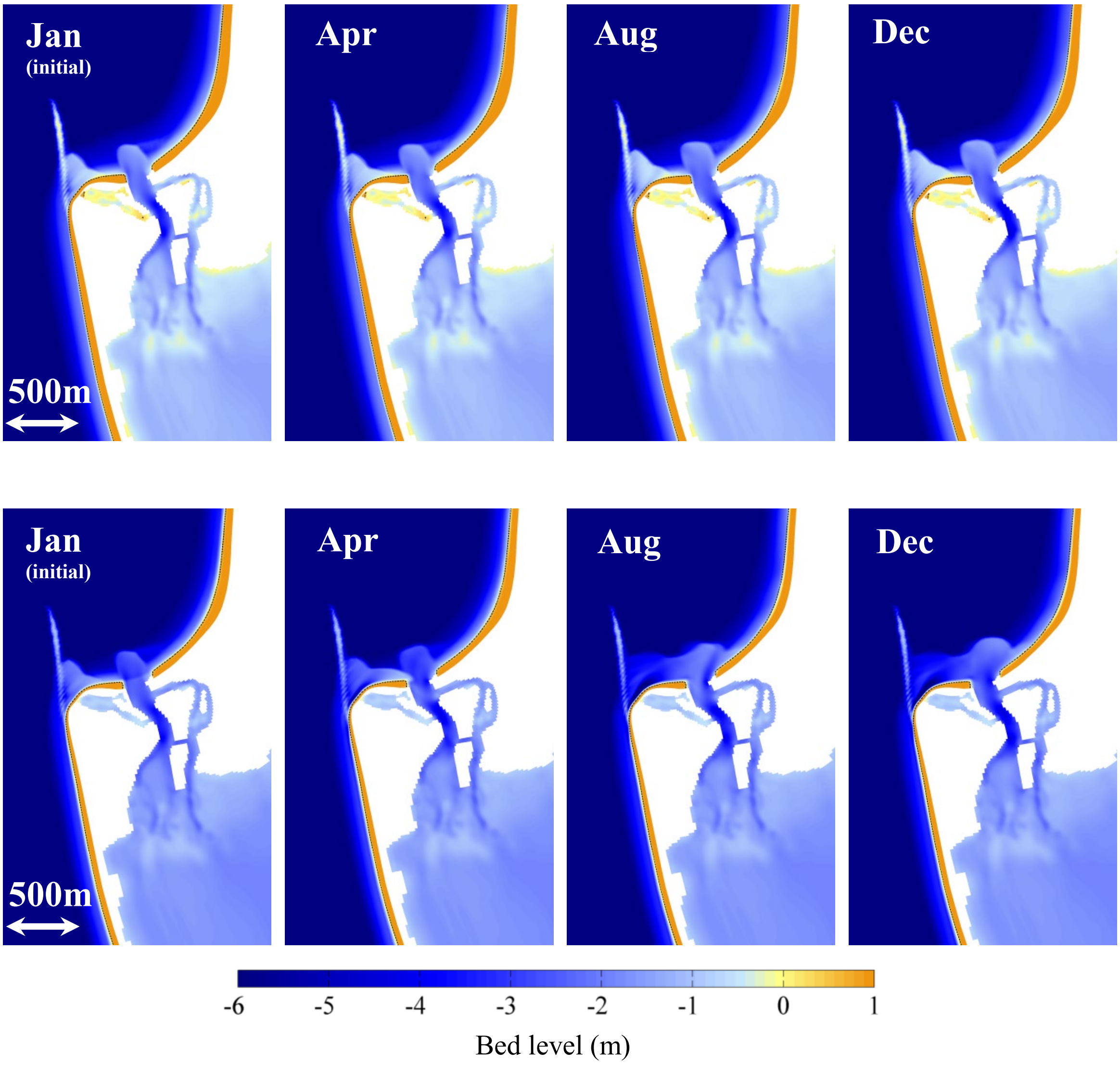

Figure S2. Modelled morphological changes for Negombo lagoon over a one year period from the process based snap-shot modelling approach (Reproduced from Duong et al., 2018[13], all rights reserved); under contemporary forcing conditions (top) and climate change modified year 2100 forcing conditions (bottom). Neither simulation indicates any significant morphological changes occurring within the annual forcing cycle. The black line indicates the initial shoreline position.

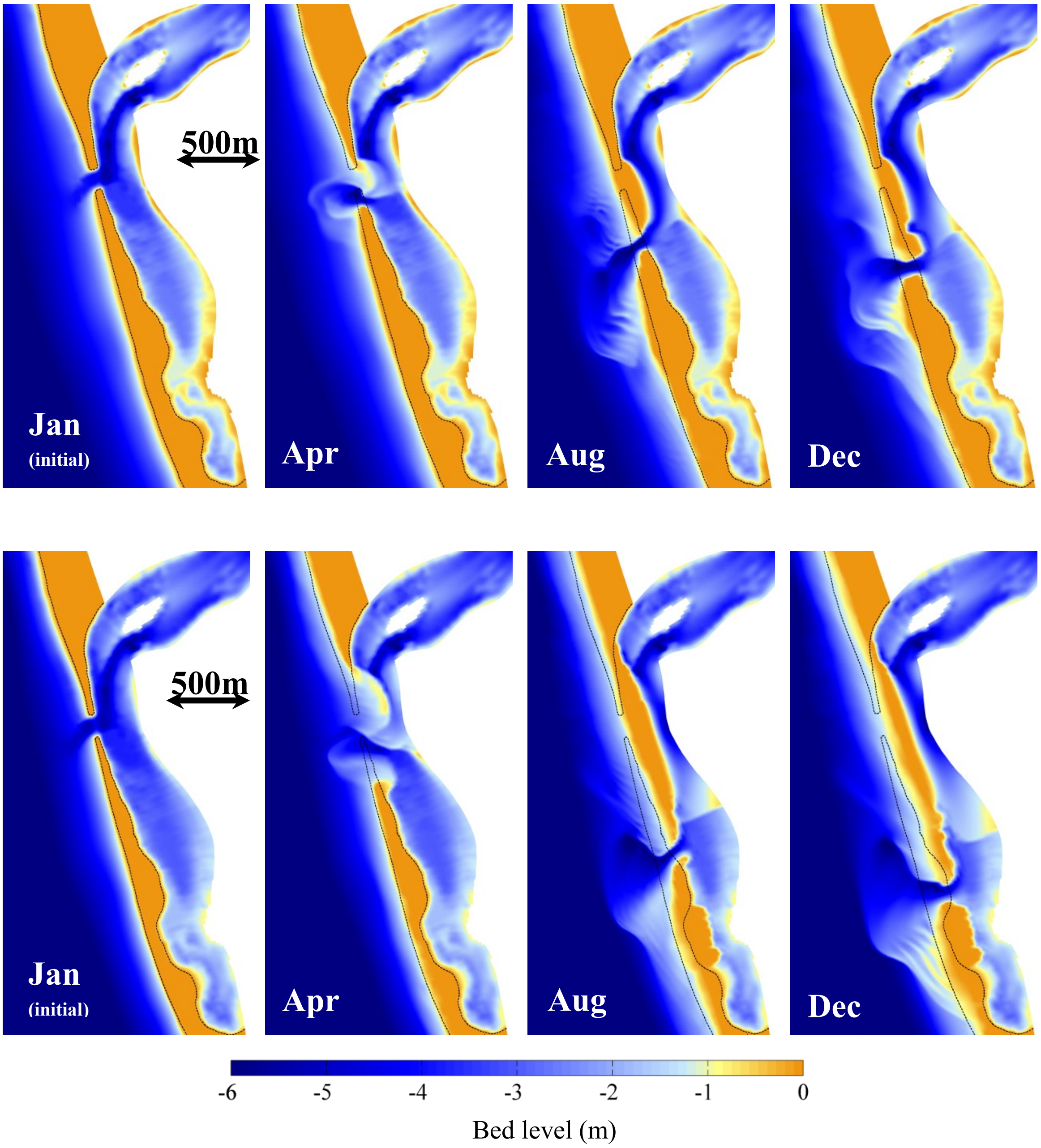

Figure S3. Modelled morphological changes for Kalutara lagoon over a one year period from the process based snap-shot modelling approach (Reproduced from Duong et al., 2018[13], all rights reserved); under contemporary forcing conditions (top) and climate change modified year 2100 forcing conditions (bottom). The black line indicates the initial shoreline position.

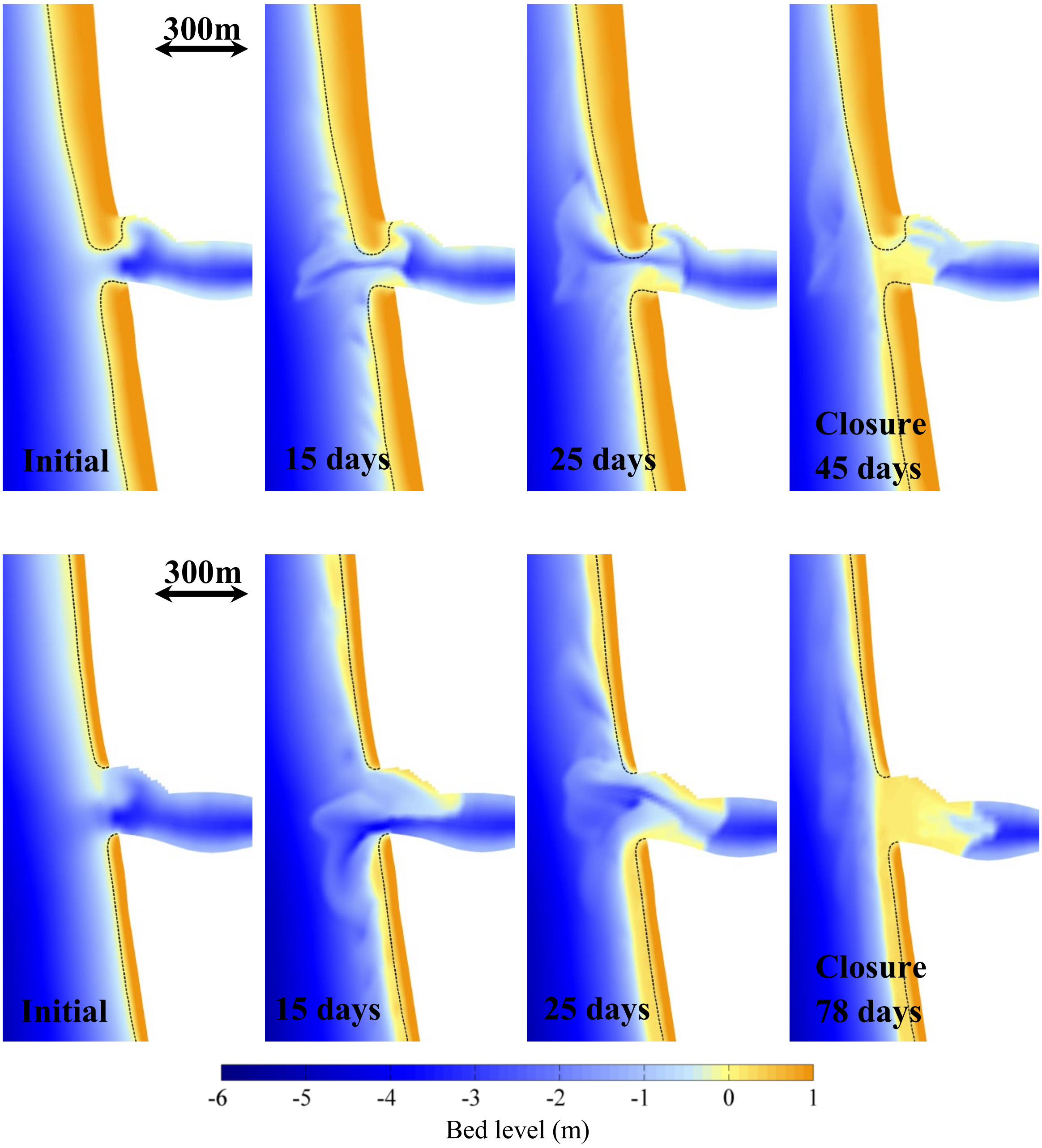

Figure S4. Modelled morphological changes for Maha Oya river until inlet closure from the process based snap-shot modelling approach (Reproduced from Duong et al., 2018[13], all rights reserved); under contemporary forcing conditions (top) and climate change modified year 2100 forcing conditions (bottom). The black line indicates the initial shoreline position.
